# Gut microbiome dysbiosis implicates the gut-bone axis in Modic changes: a metagenomic case–control study

**DOI:** 10.3389/fmicb.2025.1702357

**Published:** 2025-12-15

**Authors:** Binbin Lan, Yu Liang, Zhongxian Zhou, Jianwei Liu

**Affiliations:** Department of Spine and Osteopathy Ward, The Second People’s Hospital of Nanning, The Third Affiliated Hospital of Guangxi Medical University, Nanning, Guangxi, China

**Keywords:** Modic changes, gut microbiota, dysbiosis, low back pain, metagenomics, inflammation

## Abstract

**Introduction:**

Modic changes (MCs) are vertebral endplate lesions strongly associated with discogenic low back pain (LBP), though their pathogenesis remains poorly understood. Emerging evidence implicates gut microbial dysbiosis in systemic inflammation and musculoskeletal disorders, yet its potential role in MCs has not been investigated. This study aimed to characterize the gut microbiome in patients with MCs and identify microbial and metabolic features linked to disease severity.

**Methods:**

In a case–control study, shotgun metagenomic sequencing was performed on fecal samples from 31 patients with MCs (16 Type 1, 15 Type 2) and 25 age- and sex-matched healthy controls. Microbial community structure was assessed via alpha and beta diversity analyses. Differential taxa and predictive biomarkers were identified using linear discriminant analysis effect size (LEfSe) and Random Forest modeling. Functional potential was evaluated via Kyoto Encyclopedia of Genes and Genomes (KEGG) pathway analysis. Associations between microbial features and clinical markers (C-reactive protein [CRP], Pfirrmann grade) were also examined.

**Results:**

Patients with MCs showed significantly reduced gut microbial alpha diversity compared to controls (Chao1 index: *p* = 0.005; Shannon index: *p* = 0.034; Simpson index: *p* = 0.042), with the most pronounced reduction in Type 1 MCs. Beta diversity analysis revealed distinct microbial communities between groups (PERMANOVA, *p* = 0.001). Key discriminative taxa included *unclassified_Parabacteroides* (AUC = 0.895) and *Bacteroides uniformis* (AUC = 0.889). Metabolic pathway analysis identified 52 differentially abundant pathways, with significant enrichment of quorum sensing (*p* < 0.001) and glycerolipid metabolism (*p* < 0.001) in MC patients, both strongly correlated with elevated CRP and higher Pfirrmann grade (*p* < 0.001).

**Discussion:**

Gut microbial dysbiosis is associated with MCs, marked by reduced diversity, specific bacterial biomarkers, and altered metabolic pathways related to inflammation and tissue degeneration. These results suggest a potential role of the gut–bone axis in MC pathogenesis and highlight novel targets for diagnostic and therapeutic strategies in LBP.

## 1 Introduction

Low back pain (LBP) continues to pose a substantial global health burden, largely due to its high disability rate. Among various etiologies, vertebral endplate abnormalities identified as Modic changes (MCs) demonstrate high specificity for discogenic low back pain. In patients with LBP, the prevalence of MCs ranges from 18 to 62% (Dudli et al., 2016; Viswanathan et al., 2020), whereas epidemiological studies in asymptomatic populations report rates between 5.8 and 55.6% (Wang et al., 2012; Mok et al., 2016; Han et al., 2017; Chen L. et al., 2018). These lesions occur most frequently in the lower lumbar spine and often exhibit bilateral symmetric distribution. Type 2 MCs are the most common, followed by Type 1, while Type 3 is relatively rare.

Magnetic resonance imaging (MRI) classifies MCs into three subtypes based on signal intensity patterns: Type 1 (MC I) appears hypointense on T1-weighted images and hyperintense on T2-weighted images; Type 2 (MC II) shows hyperintensity on both T1- and T2-weighted images; and Type 3 (MC III) is hypointense on both sequences. These imaging features reflect distinct histopathological processes: MC I is associated with active inflammation in the absence of fatty marrow, MC II represents fatty degeneration, and MC III corresponds to subchondral sclerosis. Importantly, these subtypes are not static but may transition over time, reflecting a dynamic interplay between endplate pathophysiology and alterations in the bone marrow microenvironment (Herlin et al., 2018).

While our understanding of endplate inflammation has grown, it has remained confined to local processes. The potential involvement of systemic mechanisms, particularly through the gut-bone axis and microbial dysbiosis, has been largely neglected.

The gastrointestinal tract has recently emerged as a critical site for microbial influences on systemic health (Capoor et al., 2017; Zheng et al., 2020). The gut microbiota, comprising trillions of commensal microorganisms, plays vital roles in nutrient metabolism, maintenance of epithelial integrity, and regulation of immune function (Li et al., 2020; Fan and Pedersen, 2021). Beyond the gut, these microbial communities influence distant organs through several mechanisms: immunomodulation, vascular signaling, neural communication, and maintenance of bone homeostasis—with particular relevance to osteoporosis (Pellegrini et al., 2018; Zaiss et al., 2019; Cryan et al., 2020). This cross-organ communication occurs via: (1) compromised intestinal barrier permitting microbial translocation, (2) systemic circulation of pathogen-associated molecular patterns, and (3) microbial metabolites such as short-chain fatty acids that exhibit broad organ-specific effects. Notably, metabolomic studies indicate that nearly 10% of circulating metabolites in humans are microbial in origin, often referred to as “postbiotic” mediators (Ney et al., 2023; Zhang et al., 2023). Growing evidence supports the involvement of these metabolites in a range of intestinal and extra-intestinal disorders, including neurological, cardiovascular, hepatic, and musculoskeletal conditions (Biver et al., 2019).

Accumulating clinical evidence further supports the association between gut microbial dysbiosis and musculoskeletal pathologies (Aboushaala et al., 2023). For instance, altered ratios of Bacillota and Bacteroidota have been linked to osteoporosis (Wang et al., 2017), while reduced microbial diversity has been associated with ankylosing spondylitis (Cardoneanu et al., 2021). Notably, in obese populations, specific bacterial genera such as Adlercreutzia have been correlated with back pain (Dekker Nitert et al., 2020). These findings suggest that gut-derived inflammation, potentially mediated by a compromised intestinal barrier or direct bacterial invasion, may contribute to spinal degenerative processes over time.

Despite established connections between gut dysbiosis and multisystem diseases, its potential role in the development of MCs remains poorly understood. Given the inflammatory basis of MCs and the gut microbiota’s immunomodulatory functions, we hypothesize that gut dysbiosis contributes to MC pathogenesis via the gut–bone axis.

To test this hypothesis, we conducted a comprehensive comparison of the gut microbiome between patients with MCs and matched controls without MCs. This study aimed to determine whether microbial diversity, community structure, and functional potential are altered in MC patients, and to explore associations between microbial profiles and clinical disease indicators. Our findings provide new insights into the pathophysiology of MCs and may identify novel diagnostic biomarkers or therapeutic targets.

## 2 Methods

### 2.1 Study design and patient recruitment

This study was approved by the Ethics Committee of The Third Affiliated Hospital of Guangxi Medical University (Approval Code: Y2024413). Written informed consent was obtained from each participant prior to the commencement of any research-related procedures. All experimental protocols adhered to the ethical guidelines outlined in the Declaration of Helsinki.

We conducted a case–control study involving 31 patients with Modic changes (MCs group) and 25 age- and sex-matched individuals without Modic changes (non-Modic group). The MCs group consisted of 16 type 1 and 15 type 2 cases according to standardized MRI classification criteria (Modic et al., 1988). The non-Modic group comprised volunteers with no history of low back pain, no systemic comorbidities, and an absence of Modic changes, as confirmed by MRI, who were age- and sex-matched to the MCs cohort.

The exclusion criteria for both groups were as follows: (1) Structural spinal pathologies (e.g., deformities, spondylolisthesis, infectious spondylitis including tuberculosis); (2) Systemic disorders: malignancy, autoimmune diseases (e.g., rheumatoid arthritis, systemic lupus erythematosus), diabetes mellitus, inflammatory bowel disease (Crohn’s disease or ulcerative colitis), chronic kidney/liver disease, or other systemic arthritic conditions such as osteoarthritis (e.g., knee osteoarthritis); (3) Pharmacological confounders: systemic corticosteroid use, antibiotic therapy, or regular probiotic/prebiotic supplementation within 3 months prior to enrollment; (4) Pregnancy, lactation, or peri-partum status; and (5) Inflammatory arthropathies affecting the spine (e.g., ankylosing spondylitis, and psoriatic arthritis) (Chou et al., 2011; Karppinen et al., 2011; Samartzis et al., 2013; Jackson et al., 2018).

Rigorous verification of all exclusion criteria was implemented through a standardized multimodal protocol. Structural spinal pathologies were ruled out through systematic evaluation of the study-acquired lumbar MRI scans, with all images independently interpreted by two blinded radiologists. Systemic disorders, with particular attention to autoimmune conditions, were screened using a sequential approach involving standardized self-administered questionnaires that specifically inquired about diagnosed autoimmune diseases, comprehensive structured interviews, and detailed medical history review. This clinical screening was complemented by standardized fasting blood analyses. The laboratory assessments targeted glucose metabolism (fasting glucose, glycated hemoglobin), renal function (creatinine, urea), liver function (alanine aminotransferase, aspartate aminotransferase), and systemic inflammation (high-sensitivity C-reactive protein), providing objective biomarkers to corroborate the overall health status and self-reported histories. Pharmacological exposure was ascertained through a dedicated section across both questionnaires and interviews that was specifically designed to identify use of corticosteroids, antibiotics, and probiotic/prebiotic supplements within the 3-month pre-enrollment period.

### 2.2 Imaging studies

#### 2.2.1 Classification of Modic changes

**Figure 1:**
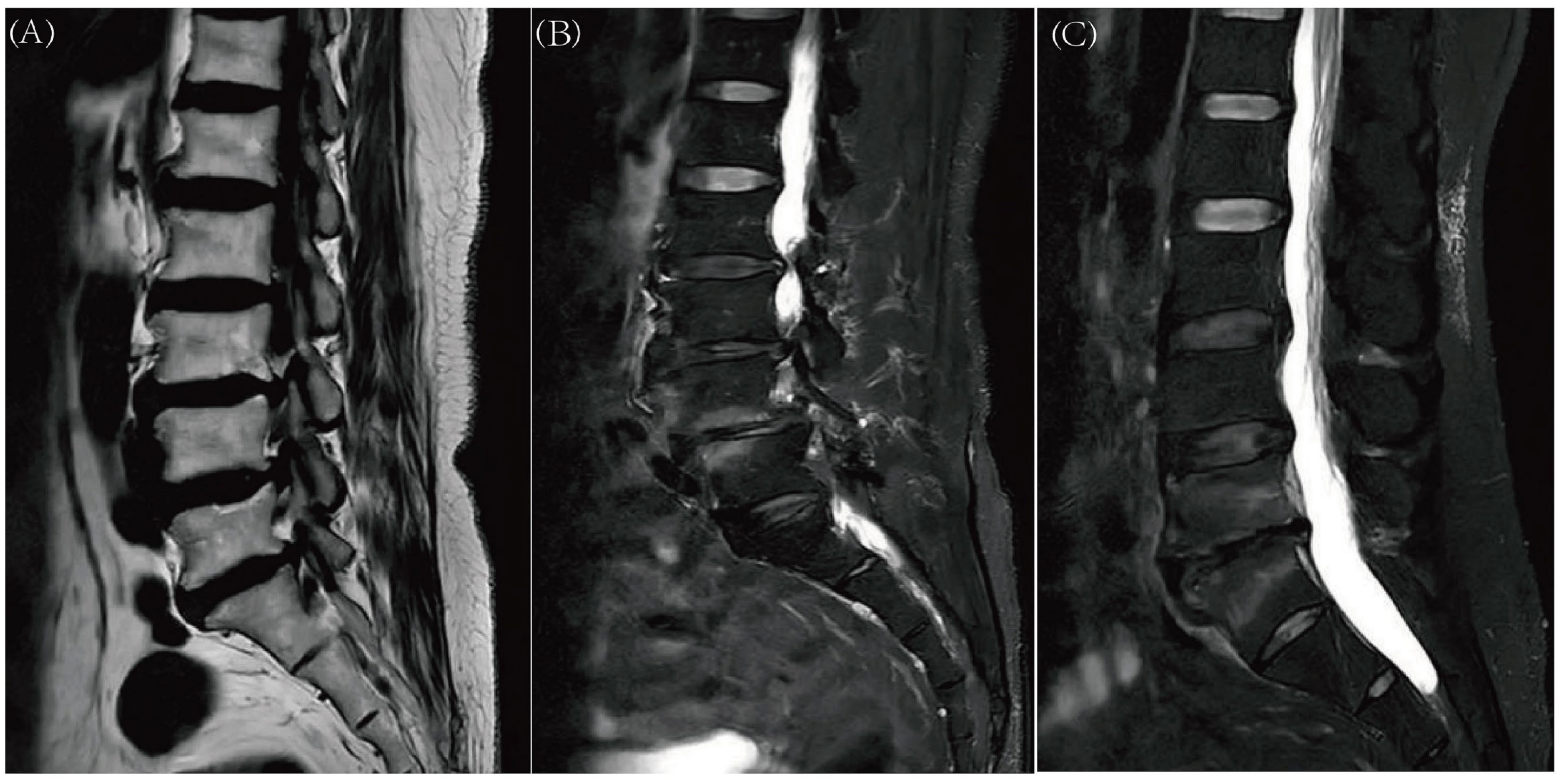
Grade of Modic changes Grade A: marrow changes of <25% of the vertebral height **(A)**. Grade B: marrow changes between 25 and 50% of the vertebral height **(B)**. Grade C: marrow changes of >50% of the vertebral height **(C)**. Classification of Modic changes type I: signal hyperintensity is also observed on fat-suppressed T2-weighted MRI **(B,C)**. Classification of Modic changes type II: signal hyperintensity is identified on T1-weighted MRI **(A)**.

All MRI assessments were performed independently by two experienced clinicians who were blinded to the clinical grouping of the participants. Two experienced clinicians independently evaluated all parameters, with particular focus on the radiological characteristics defining the type and grade of MCs. In instances where initial assessments diverged, the observers conducted a joint re-evaluation. Persistent discrepancies were resolved by adjudication from a senior professor. Based on established criteria (Modic et al., 1988), MCs were categorized into three distinct types according to their signal characteristics on MRI: Type 1 lesions exhibit low signal intensity on T1-weighted images and high signal intensity on T2-weighted images. Type 2 lesions demonstrate high signal intensity on both T1- and T2-weighted images. Conversely, Type 3 lesions are characterized by low signal intensity on both T1- and T2-weighted sequences. This study exclusively focused on Modic Type 1 and Type 2 changes (Figure 1).

#### 2.2.2 Grading of MCs

The severity of MCs was graded into three levels (A, B, C) based on the vertical extent of bone marrow involvement within the vertebral body, measured on sagittal MRI slices according to standard methodology (Udby et al., 2022). Grade A represents involvement affecting less than 25% of the vertebral body height. Grade B indicates involvement spanning 25 to 50% of the vertebral height. Grade C denotes extensive involvement encompassing more than 50% of the vertebral height (Figure 1).

#### 2.2.3 Assessment of disc degeneration (Pfirrmann grade)

The severity of intervertebral disc degeneration adjacent to Modic changes was evaluated on T2-weighted MRI according to the Pfirrmann classification system. This system categorizes degenerative changes into five distinct grades based on disc morphology, signal intensity, and structural integrity. Grade I discs exhibit a homogeneous bright hyperintense signal with preserved disc height. Grade II discs are inhomogeneous yet maintain a hyperintense signal intensity, a clear demarcation between the nucleus pulposus and annulus fibrosus, and normal height. Progression to Grade III is marked by inhomogeneous intermediate (gray) signal intensity, loss of the clear nuclear-annular distinction, and normal or mildly reduced disc height. Grade IV discs show inhomogeneous hypointense (dark gray) signal, complete obliteration of the nuclear-annular boundary, and normal or moderately decreased height. The most advanced degeneration, Grade V, is characterized by a hypointense black signal, absence of internal architecture, and collapsed disc space (Pfirrmann et al., 2001).

### 2.3 Metagenomic sequencing and bioinformatics analysis

#### 2.3.1 Fecal sample collection and DNA extraction

Fecal specimens were collected at The Third Affiliated Hospital of Guangxi Medical University. Participants received standardized instructions and collection kits containing DNA/RNA Shield stabilizer tubes to minimize pre-analytical variation. All samples were cryopreserved at −80 °C within two hours of collection. Specimens were subsequently transported on dry ice to Cosmos Wisdom Technology Co., Ltd. (Hangzhou, China) for nucleic acid extraction using established protocols (Wu et al., 2019). Total genomic DNA was isolated from 0.2 g of fecal samples with the FastPure Stool DNA Isolation Kit (Magnetic Bead) (Vazyme Biotech, Nanjing, China), following the protocols provided by the manufacturer. DNA concentration was measured using a Synergy HTX Multi-Mode Microplate Reader (BioTek Instruments, Inc., Winooski, VT, United States). Purity was evaluated based on A260/A280 and A260/A230 ratios obtained with a NanoDrop 2000 spectrophotometer (Thermo Fisher Scientific, Wilmington, DE, United States). Integrity was assessed by electrophoretic separation on 1% agarose gels. Only those samples meeting the following criteria were included in subsequent sequencing: DNA concentration exceeding 10 ng/μL, A260/A280 ratios between 1.8 and 2.0, and the presence of distinct high-molecular-weight bands upon gel visualization.

#### 2.3.2 Library preparation and sequencing

DNA extracts were fragmented to an average size of approximately 350 bp using a Covaris M220 focused-ultrasonicator (Covaris, Woburn, MA, United States; distributed by Gene Company Limited, China). Paired-end libraries were prepared with the NEXTFLEX Rapid DNA-Seq Kit (Bioo Scientific, Austin, TX, United States). Subsequent metagenomic sequencing was carried out on an Illumina NovaSeq X Plus system (Illumina Inc., San Diego, CA, United States) employing the NovaSeq X Series 25B Reagent Kit at Cosmos Wisdom Technology Co., Ltd. (Hangzhou, China), in strict accordance with the manufacturer’s instructions. The sequencing depth was targeted at a minimum of 20 million high-quality paired-end reads (150 bp) per sample to ensure sufficient coverage for downstream analysis.

#### 2.3.3 Processing of metagenomic sequencing data

Bioinformatic analysis was conducted using the Cosmos Wisdom free online platform. Briefly, raw sequencing reads were processed to remove adapter sequences, and low-quality reads (length < 50 bp or average Phred quality score <Q20) were filtered using fastp (Chen S. et al., 2018) (version 0.20.0). Reads aligning to the human reference genome (hg38) were identified using BWA-MEM (Li and Durbin, 2009) (version 0.7.17) and subsequently removed, including both the aligned read and its mate pair. Quality-filtered reads were *de novo* assembled into contigs using MEGAHIT (Li et al., 2015) (version 1.1.2). Contigs ≥300 bp in length were retained for downstream analysis. Open reading frames (ORFs) were predicted from these contigs using Prodigal (Hyatt et al., 2010) (version 2.6.3), and ORFs ≥ 100 bp were extracted. A non-redundant gene catalog was constructed from the predicted ORFs using CD-HIT (Fu et al., 2012) (version 4.7) with clustering thresholds of 90% sequence identity and 90% coverage. Gene abundance, expressed as reads per kilobase per million mapped reads (RPKM), was quantified for each sample by aligning the quality-filtered reads back to the non-redundant gene catalog using SOAPaligner/soap2 (Li et al., 2008) (version 2.21), requiring ≥95% sequence identity.

#### 2.3.4 Taxonomic and functional annotation

Taxonomic annotation of the non-redundant genes was performed by aligning them against the NCBI non-redundant protein (NR) database using DIAMOND (Buchfink et al., 2015) (version 2.0.13) with a maximum E-value cutoff of 1e-5. The best-hit taxonomic assignment for each gene was determined based on these alignments. Functional annotation was similarly conducted against the following databases using DIAMOND (E-value ≤ 1e-5) to assign functional categories: Gene Ontology (GO), Kyoto Encyclopedia of Genes and Genomes (KEGG), Evolutionary Genealogy of Genes: Non-supervised Orthologous Groups (eggNOG), Carbohydrate-Active enZYmes (CAZy), Comprehensive Antibiotic Resistance Database (CARD), and Pathogen-Host Interactions (PHI) database.

### 2.4 Statistical analysis

All statistical analyses were conducted using R software (version 4.4.3). A false discovery rate (FDR)-adjusted *p*-value threshold of < 0.05 was applied to define statistical significance, unless explicitly stated otherwise.

We first compared differential abundance across taxonomic ranks (phylum, genus, species), functional KEGG pathways, and gene-level features between the Modic I/II and Non-Modic groups using the Kruskal–Wallis test on RPKM-normalized data. The resulting *p*-values within each taxonomic or functional category were corrected for multiple testing using the Benjamini–Hochberg false discovery rate (FDR) procedure. Effect sizes were reported using Cliff’s delta for continuous variables and Cramer’s V for categorical variables.

To compare alpha diversity (Chao1, Shannon, and Simpson indices), we conducted Wilcoxon rank-sum tests for multiple group pairs: Modic I/II vs. Controls, Modic I vs. Controls, Modic II vs. Controls, and Modic I vs. Modic II. *p*-values from these tests were adjusted for multiple comparisons using the Benjamini–Hochberg FDR method. Beta diversity, based on Bray–Curtis dissimilarity matrices at the phylum level, was evaluated with permutational multivariate analysis of variance (PERMANOVA) with 999 permutations for the same set of comparisons.

To identify differentially abundant taxa between Modic and non-Modic groups, we performed linear discriminant analysis effect size (LEfSe) analysis. This analysis first identified significant taxa via the Kruskal–Wallis test (*p* < 0.05), then estimated the effect size of each feature using linear discriminant analysis (LDA), retaining those with LDA scores > 2.0. The top 10 taxa from each group were selected, resulting in 20 candidate species-level biomarkers. These were further evaluated with receiver operating characteristic (ROC) curve analysis, where an area under the curve (AUC) > 0.7 was considered indicative of good discriminatory performance.

For functional profiling, the same LEfSe method was applied to identify enriched KEGG pathways (Level 3) with LDA > 2.0. Significantly enriched pathways were visualized in a heatmap and subjected to ROC curve analysis. The top 10 pathways based on AUC values were selected, and their abundances were compared between Modic and non-Modic groups. Associations between these pathways and clinical variables (e.g., CRP, Pfirrmann grade) were assessed using Spearman’s rank correlation, with correlation coefficients (*ρ*) and FDR-adjusted *p*-values reported.

We also employed Random Forest modeling to identify microbial biomarkers associated with Modic change severity. The model incorporated all species-level taxa with prevalence greater than 10%. Features were ranked by mean decrease accuracy (MDA), and the top ten species with MDA > 1.5 were retained as candidate biomarkers. Their abundance profiles across severity grades were visualized using a stacked bar plot, and correlations with Modic grades were further validated via Spearman’s analysis.

All figures were generated using the ggplot2, pheatmap, and vegan packages in R.

## 3 Results

### 3.1 Clinical features in Modic and non-Modic groups

This study enrolled 56 participants: 31 with Modic changes and 25 without. No significant differences existed in baseline characteristics including age, gender distribution, or body mass index (all *p* > 0.05), confirming group comparability. Serum high-sensitivity C-reactive protein (hs-CRP) levels were significantly higher in the Modic change group versus controls (17.0 ± 10.7 mg/L vs. 4.5 ± 6.3 mg/L; *p* < 0.001). Pfirrmann grade distributions differed significantly between groups (*p* < 0.001). The Modic change group demonstrated advanced-stage degeneration (grades III-V), with 35.5% grade III (moderate), 16.1% grade IV (severe), and 48.4% grade V (collapse). In contrast, controls primarily exhibited mild-to-moderate degeneration (grades II–III), comprising 92% of this group. Specifically, 4.0% had grade I (normal), 40.0% grade II (mild), and 52.0% grade III (moderate) degeneration, with no grade V cases observed (Table 1).

**Table 1:** Comparative demographic and clinical profiles with vs. without Modic changes.

Variable	Modic (*n* = 31)	Non-Modic (*n* = 25)	*P*-value
Age (years)^§^	70.3 ± 9.8	69.3 ± 9.7	0.703
BMI (kg/m^2^)^§^	24.0 ± 1.9	23.3 ± 1.9	0.245
Gender^a^			>0.999
Male	14 (45.2%)	12 (48.0%)	
Female	17 (54.8%)	13 (52.0%)	
Pfirrmann Grade^a^			<0.001
I	0 (0.0%)	1 (4.0%)	
II	0 (0.0%)	10 (40.0%)	
III	11 (35.5%)	13 (52.0%)	
IV	5 (16.1%)	1 (4.0%)	
V	15 (48.4%)	0 (0.0%)	
MCs Grade^a^			<0.001
None Modic change	0 (0.0%)	25 (100.0%)	
Grade A	9 (29.0%)	0 (0.0%)	
Grade B	7 (22.6%)	0 (0.0%)	
Grade C	15 (48.4%)	0 (0.0%)	
hs-CRP (mg/L)^§^	17.0 ± 10.7	4.5 ± 6.3	<0.001

Continuous variables (§) were analyzed by Student’s *t*-test. Categorical variables (ª) were tested by Fisher’s exact test. §, mean ± SD; ª, *n* (%). BMI, Body Mass Index; MCs Grade, Modic Changes Grade; hs-CRP, high-sensitivity C-reactive protein; SD, standard deviation.

Comparative analysis of Modic type I (*n* = 16), type II (*n* = 15), and non-Modic (*n* = 25) cohorts revealed significant differences in inflammatory and degenerative profiles. Serum CRP levels were markedly elevated in both Modic subtypes compared to non-Modic controls (Modic I: 20.2 ± 11.0 mg/L; Modic II: 13.6 ± 9.6 mg/L; non-Modic: 4.5 ± 6.3 mg/L; both *p* < 0.01 vs. non-Modic group), though no significant difference existed between subtypes (*p* = 0.110). Disc degeneration severity differed substantially, with grade V collapse prevalent in 62.5% of Modic type I, 33.3% of Modic type II, and absent in non-Modic subjects, who predominantly exhibited mild-to-moderate degeneration (92% grades II–III). Both Modic subtypes exhibited significantly worse degeneration than non-Modic controls (I vs. non-Modic: *p* < 0.001; II vs. non-Modic: *p* < 0.001). No intergroup differences were observed in age, body mass index (BMI), or gender distribution (all *p* > 0.05) (Table 2).

**Table 2:** Comparative demographic and clinical profiles among Modic type I, type II, and non-Modic groups.

Variable	Modic I (*n* = 16)	Modic II (*n* = 15)	Non-Modic (*n* = 25)	*p*-value (I vs II)	*p*-value (I vs Non)	*p*-value (II vs Non)
Age (years)^§^	72.9 ± 9.7	67.6 ± 9.3	69.3 ± 9.7	0.148	0.335	0.780
BMI (kg/m^2^)^§^	23.9 ± 2.3	24.0 ± 1.6	23.3 ± 1.9	0.635	0.539	0.276
Gender^a^				1.000	1.000	1.000
Male	7 (43.8%)	7 (46.7%)	12 (48.0%)			
Female	9 (56.3%)	8 (53.3%)	13 (52.0%)			
Pfirrmann Grade^a^				0.263	<0.001	<0.001
I	0 (0.0%)	0 (0.0%)	1 (4.0%)			
II	0 (0.0%)	0 (0.0%)	10 (40.0%)			
III	4 (25.0%)	7 (46.7%)	13 (52.0%)			
IV	2 (12.5%)	3 (20.0%)	1 (4.0%)			
V	10 (62.5%)	5 (33.3%)	0 (0.0%)			
MC Grade^a^				0.063	<0.001	<0.001
None Modic change	0 (0.0%)	0 (0.0%)	25 (100.0%)			
Grade A	3 (18.8%)	6 (40.0%)	0 (0.0%)			
Grade B	2 (12.5%)	5 (33.3%)	0 (0.0%)			
Grade C	11 (68.8%)	4 (26.7%)	0 (0.0%)			
CRP (mg/L)^§^	20.2 ± 11.0	13.6 ± 9.6	4.5 ± 6.3	0.110	<0.001	0.007

Continuous variables (§) were analyzed by Student’s *t*-test. Categorical variables (ª) were tested by Fisher’s exact test. §, mean ± SD; ª, *n* (%). Comparisons: I vs. II (Modic Type 1 vs. Type 2), I vs. Non (Modic Type 1 vs. Non-Modic Controls), II vs. Non (Modic Type 2 vs. Non-Modic Controls). BMI, Body Mass Index; MCs Grade, Modic Changes Grade; hs-CRP, high-sensitivity C-reactive protein; SD, standard deviation.

### 3.2 Diversity of the gut microbiome: comparison of subjects with Modic changes and controls

**Figure 2:**
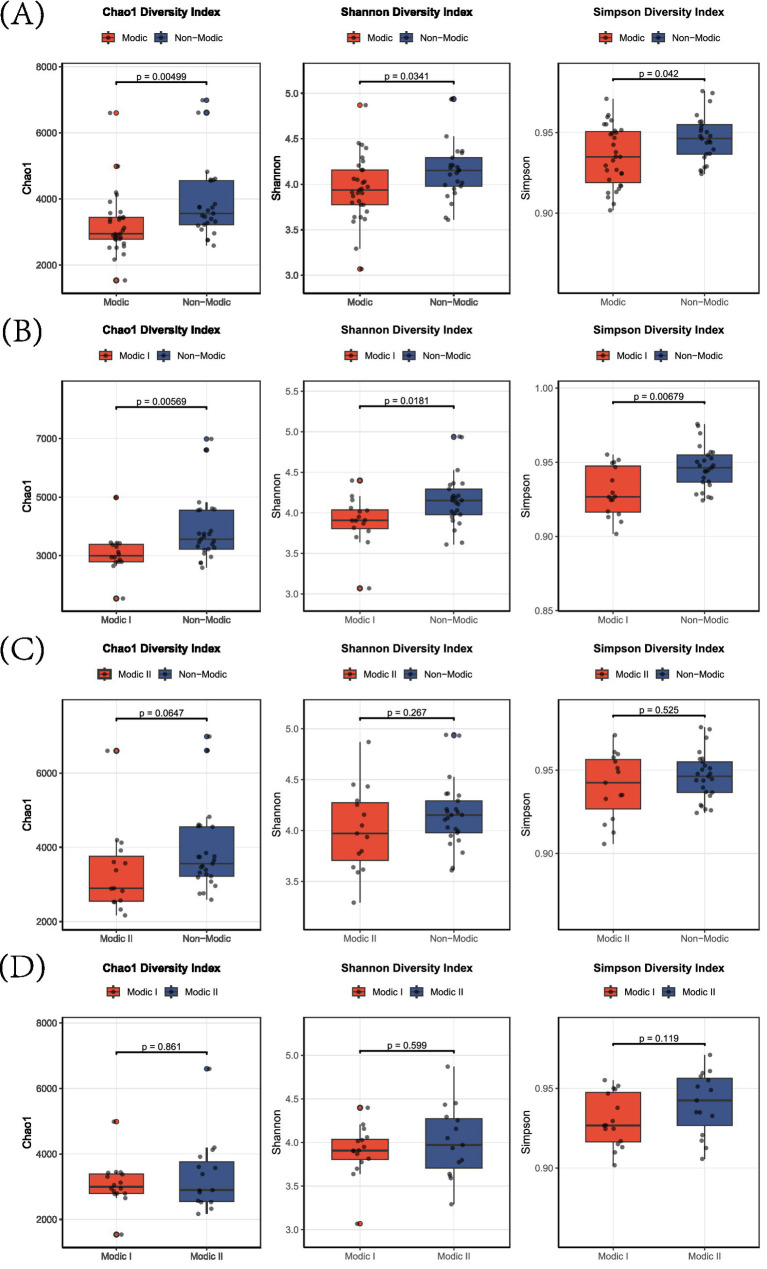
Alpha-diversity indices in Modic change subtypes. Box plots showing Chao1 (species richness), Shannon (diversity), and Simpson (dominance) indices for: **(A)** Modic I/II vs. non-Modic, **(B)** Modic I vs. non-Modic, **(C)** Modic II vs. non-Modic, and **(D)** Modic I vs. Modic II subjects.

Alpha diversity was significantly reduced in patients with combined Modic changes (Types I + II) compared to non-Modic controls (Chao1 index: *p* = 0.005; Shannon index: *p* = 0.034; Simpson index: *p* = 0.042; Figure 2A). This reduction was primarily driven by the Modic Type I subgroup, where diversity indices were markedly lower than in non-Modic subjects (Chao1: *p* = 0.006; Shannon: *p* = 0.018; Simpson: *p* = 0.007; Figure 2B). In contrast, for Modic Type II, only non-significant trends toward higher diversity in controls were observed (Chao1: *p* = 0.065; Shannon: *p* = 0.267; Simpson: *p* = 0.525; Figure 2C). Direct comparison between the two subtypes revealed no significant differences across all indices (all *p* > 0.05; Figure 2D).

**Figure 3:**
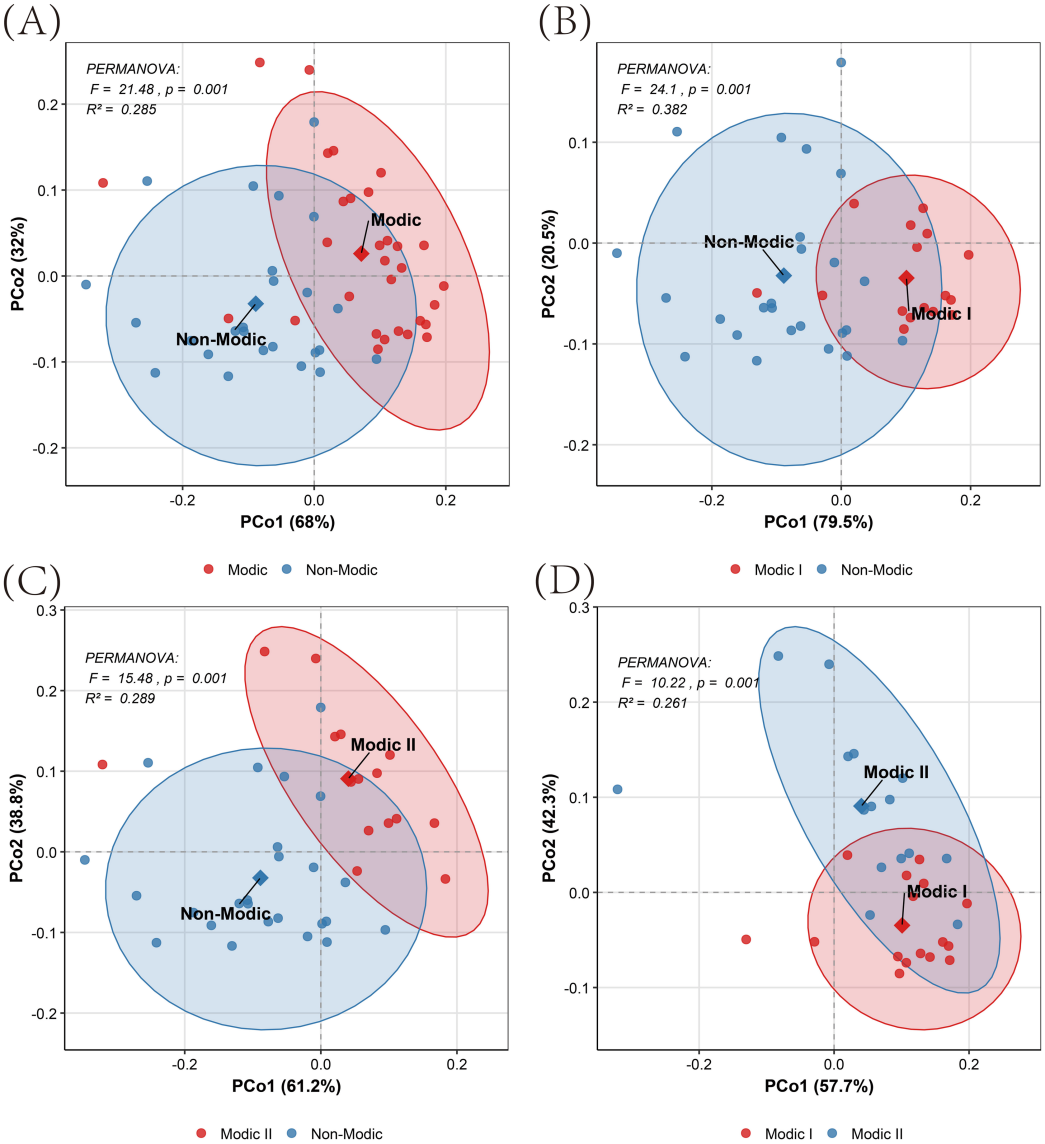
Principal coordinates analysis (PCoA) of microbial beta diversity. **(A)** Modic I/II vs. non-Modic, **(B)** Modic I vs. non-Modic, **(C)** Modic II vs. non-Modic, **(D)** Modic I vs. Modic II. Points represent individual samples; ellipses indicate 95% confidence intervals; diamonds denote group centroids. PERMANOVA statistics (*F*/*p*/*R*^2^) are reported within each panel.

Beta diversity analysis, assessed by PERMANOVA, further confirmed robust microbial community dissimilarities across all group pairs (*p* = 0.001). The combined Modic cohort substantially diverged from non-Modic controls (*F* = 21.48, *R*^2^ = 0.285; Figure 3A). Stratified analysis showed particularly pronounced effects in both Modic I vs. non-Modic (*F* = 24.1, *R*^2^ = 0.382; Figure 3B) and Modic II vs. non-Modic (*F* = 15.48, *R*^2^ = 0.289; Figure 3C) comparisons. Notably, a significant community difference was also evident between Modic I and II subtypes themselves (*F* = 10.22, *R*^2^ = 0.261; Figure 3D). The *R*^2^ values, indicating interpreted variance, ranged from 0.261 to 0.382, corresponding to medium-large effect sizes. Of clinical relevance, the Modic Type I subgroup exhibited the most severe microbial differentiation (R^2^ = 0.382), underscoring its uniquely dysbiotic state.

### 3.3 Discriminative microbial biomarkers identified by LEfSe and validated by ROC analysis

**Figure 4:**
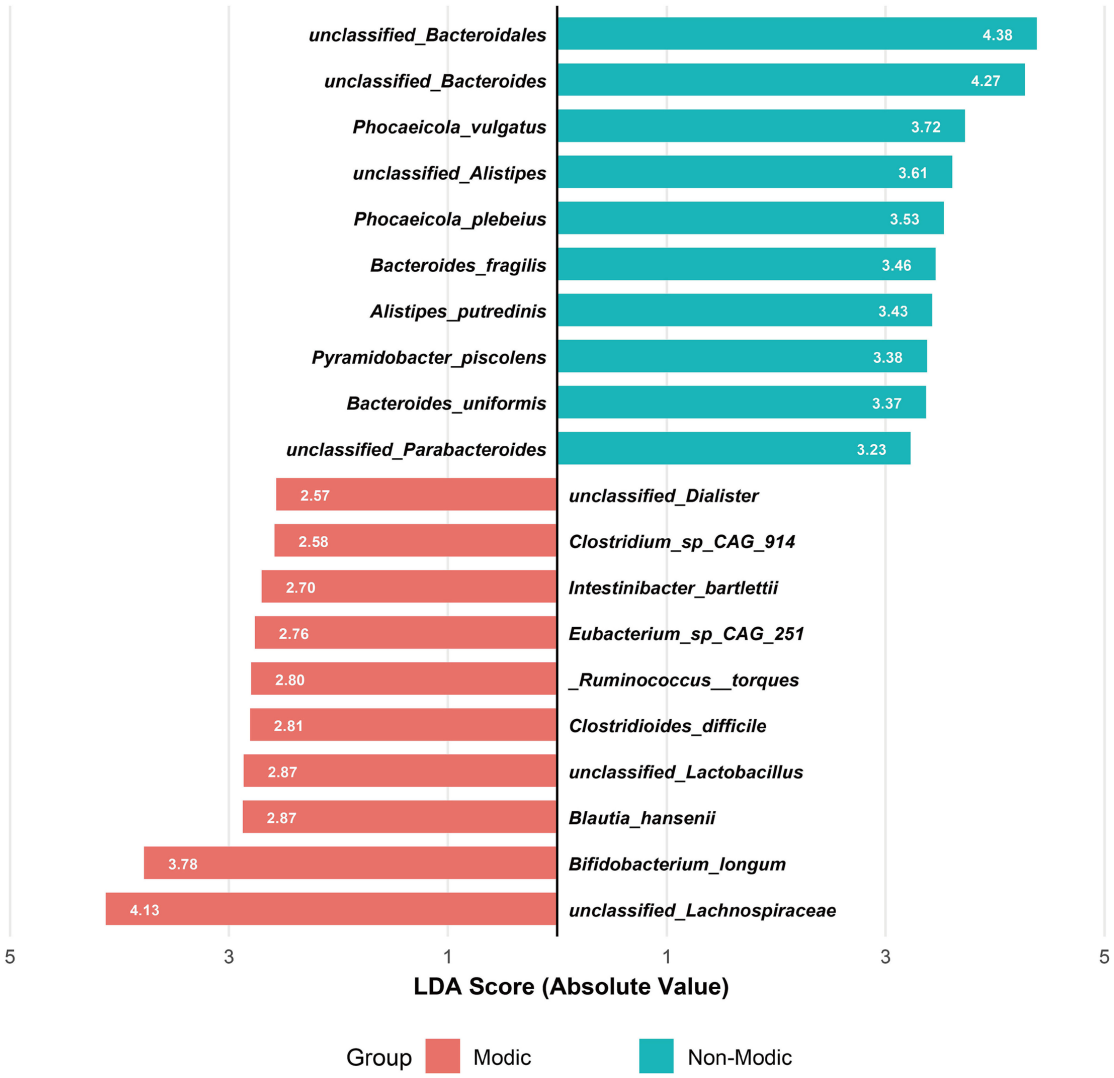
Linear discriminant analysis (LDA) of gut microbiota composition associated with Modic changes. Bidirectional LDA plot showing the top 10 discriminative species per group (LDA > 2, *p* < 0.05) between Modic type I/II patients (*n* = 31) and healthy controls (*n* = 25), revealing opposing microbial signatures characterized by Bacteroidetes dominance in controls versus Firmicutes enrichment in Modic changes.

LEfSe analysis identified the top 10 bacterial species with the highest Linear Discriminant Analysis (LDA) scores in the MC and non-MC groups, respectively, yielding a total of 20 potential biomarkers (Figure 4). These species showed significant differential abundance between patients with MC and non-MC controls (LDA score >2.0, *p* < 0.05).

**Figure 5:**
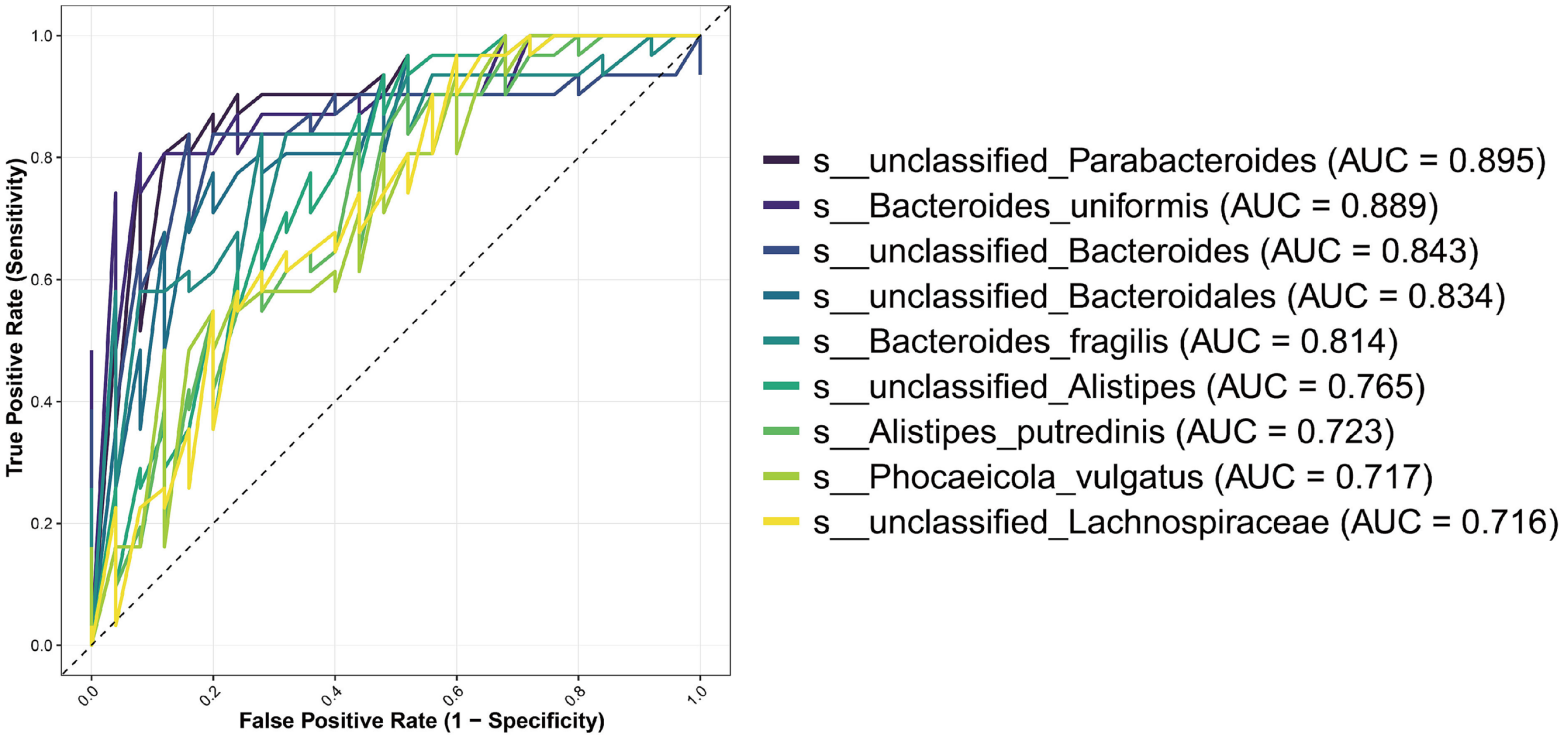
ROC curves of the most discriminative microbial species for Modic changes. Twenty candidate species were initially identified by LEfSe analysis (LDA score > 2, *p* < 0.05). This figure presents the receiver operating characteristic (ROC) curves for the subset of nine species that achieved an area under the curve (AUC) value greater than 0.7, indicating good to excellent diagnostic capability for distinguishing the Modic changes (MCs) group from non-Modic controls.

To assess the diagnostic potential of these 20 candidate biomarkers, we performed Receiver Operating Characteristic (ROC) curve analysis and calculated the Area Under the Curve (AUC) for each species (Figure 5). Nine species exhibited AUC values greater than 0.7, indicating strong discriminatory power. The top-performing biomarkers were *unclassified_Parabacteroides* (AUC = 0.895), *Bacteroides uniformis* (AUC = 0.889), *unclassified_Bacteroides* (AUC = 0.843), *unclassified_Bacteroidales* (AUC = 0.834), and *Bacteroides fragilis* (AUC = 0.814).

### 3.4 Microbial composition and predictive biomarkers associated with MCs severity

**Figure 6:**
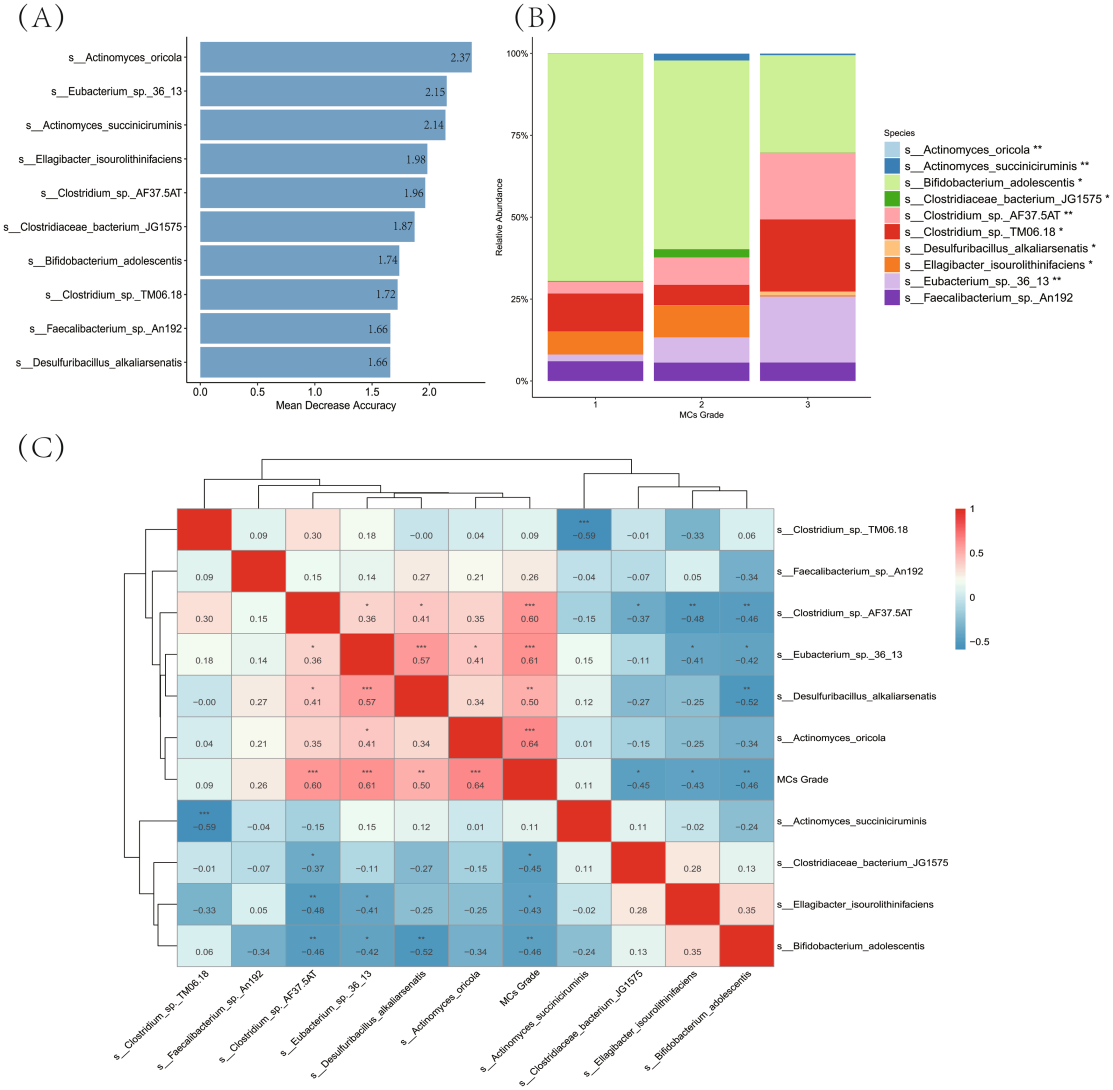
Identification of gut microbiota biomarkers associated with MCs severity. **(A)** Random forest feature importance ranking: the horizontal bar plot indicate mean decrease accuracy values for the top 10 predictive species (filtered by >10% prevalence), arranged in descending importance from top to bottom. **(B)** Compositional shifts across MCs grades: stacked bars show relative abundance (%) of key taxa, with color-coding representing bacterial species. Statistical significance markers: ***p < 0.001; **p < 0.01; *p < 0.05 (Kruskal-Wallis test). **(C)** Correlation analysis with disease severity: heatmap colors represent Spearman’s *ρ* values (red: positive; blue: negative), displaying only significant correlations (FDR *p* < 0.05). Dendrograms indicate hierarchical clustering relationships.

Random Forest modeling identified ten bacterial features at the species level with high predictive value for MCs severity (Figure 6A). These features were selected from all species-level taxa following the removal of low-abundance taxa (i.e., retaining only those present in >10% of samples)—ensuring all analyzed targets were defined and filtered at the species level. Specifically, *Actinomyces oricola* exhibited the highest feature importance (Mean Decrease Accuracy = 2.37), followed by *Eubacterium* sp. 36_13 (2.15) and *Actinomyces succiniciruminis* (2.14), with *Faecalibacterium* sp. An192 ranking ninth (1.66).

Compositional analysis of the gut microbiota across MCs grades—focusing on these ten key predictive biomarkers—revealed significant shifts (Figure 6B). Among these biomarkers, *Bifidobacterium adolescentis* dominated at Grade 1 (relative abundance: 69.4%) but decreased substantially at Grade 3 (29.8%, *p* < 0.001). In contrast, several Clostridium-related taxa showed progressive enrichment: *Clostridium* sp. TM06.18 increased from 11.6% (Grade 1) to 22.0% (Grade 3; *p* < 0.05), while *Clostridium* sp. AF37.5AT expanded from 3.6 to 20.3% (*p* < 0.05). *Eubacterium* sp. 36_13 exhibited a marked increase in abundance, with levels rising to 7.7% at Grade 2 and 20.2% at Grade 3 (*p* < 0.01). Notably, *Faecalibacterium* sp. An192 maintained consistent abundance across all grades (5.6–6.0%, *p* > 0.05).

Spearman correlation analysis further elucidated the associations between these top predictive biomarkers and MCs severity (Figure 6C). Among them, *Actinomyces oricola* demonstrated the strongest positive correlation with MCs grade (*ρ* = 0.64, *p* < 0.001), followed by *Eubacterium* sp. 36_13 (ρ = 0.61, *p* < 0.001) and *Clostridium* sp. AF37.5AT (ρ = 0.60, *p* < 0.001). *Desulfuribacillus alkaliarsenatis* also showed a significant positive correlation (ρ = 0.50, *p* < 0.01). In contrast, significant negative correlations were observed for *Ellagibacter isourolithinifaciens* (ρ = −0.43, *p* < 0.05), Clostridiaceae bacterium JG1575 (*ρ* = −0.45, *p* < 0.05), and *Bifidobacterium adolescentis* (ρ = −0.46, *p* < 0.05). Notably, *Faecalibacterium* sp. An192 showed a positive but non-significant correlation with disease grade (ρ = 0.26, *p* > 0.05), consistent with its stable abundance across severity levels described above.

### 3.5 Differential analysis of intestinal flora metabolic pathways and their discriminative capacity in Modic changes

**Figure 7:**
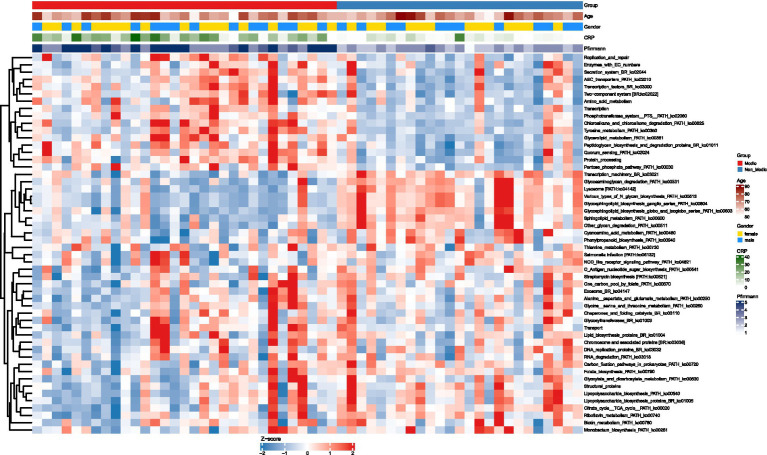
KEGG pathway (LDA > 2, *p* < 0.05) abundance patterns in Modic vs. non-Modic groups. Heatmap of significantly differentially abundant KEGG level 3 pathways (LDA > 2, adjusted *p* < 0.05) between Modic and non-Modic groups. Pathway abundances are Z-score normalized (blue = low, red = high). Rows = KEGG pathways; columns = samples, ordered by group (Modic first) without clustering. Pathways are hierarchically clustered (right dendrogram). Top annotations show clinical covariates: group (Modic = red, Non-Modic = blue), age (white to dark red = increasing age), gender (female = gold, male = blue), CRP (white to dark green = increasing levels), and Pfirrmann grade (light to dark blue = increasing degenerative grade).

Using LEfSe analysis, intergroup comparison of microbial metabolic functions revealed 52 KEGG pathways (Level 3) with significant differential abundance (LDA > 2, *p* < 0.05; see Supplementary material for the complete list) between subjects with Modic changes (MCs) and controls, underscoring systemic functional alterations in the gut microbiome associated with MCs. Unsupervised clustering analysis based on the 52 differential pathways clearly revealed distinct abundance patterns between the MCs and non-Modic groups in the heatmap (Figure 7), providing visual evidence of systemic metabolic differences (Table 3).

**Table 3:** Bacterial composition percentages from stacked bar plot by MCs grade.

Bacterial species	MCs Grade		
	Grade 1	Grade 2	Grade 3
*Actinomyces_oricola*	0.0%	0.1%	0.1%
*Actinomyces_succiniciruminis*	0.1%	2.0%	0.4%
*Bifidobacterium_adolescentis*	69.4%	57.6%	29.8%
*Clostridiaceae_bacterium*_JG1575	0.2%	2.5%	0.1%
*Clostridium*_*sp.*_AF37.5AT	3.6%	8.4%	20.3%
*Clostridium*_*sp.*_TM06.18	11.6%	6.2%	22.0%
*Desulfuribacillus_alkaliarsenatis*	0.1%	0.1%	1.2%
*Ellagibacter_isourolithinifaciens*	6.9%	9.7%	0.3%
*Eubacterium*_*sp.*_36_13	2.1%	7.7%	20.2%
*Faecalibacterium*_*sp.*_An192	6.0%	5.6%	5.6%

Values represent relative abundance of each bacterial species as a percentage of the total analyzed bacterial community (visualized in Figure 6B). Sums to 100% for each grade represent complete community composition.

**Figure 8:**
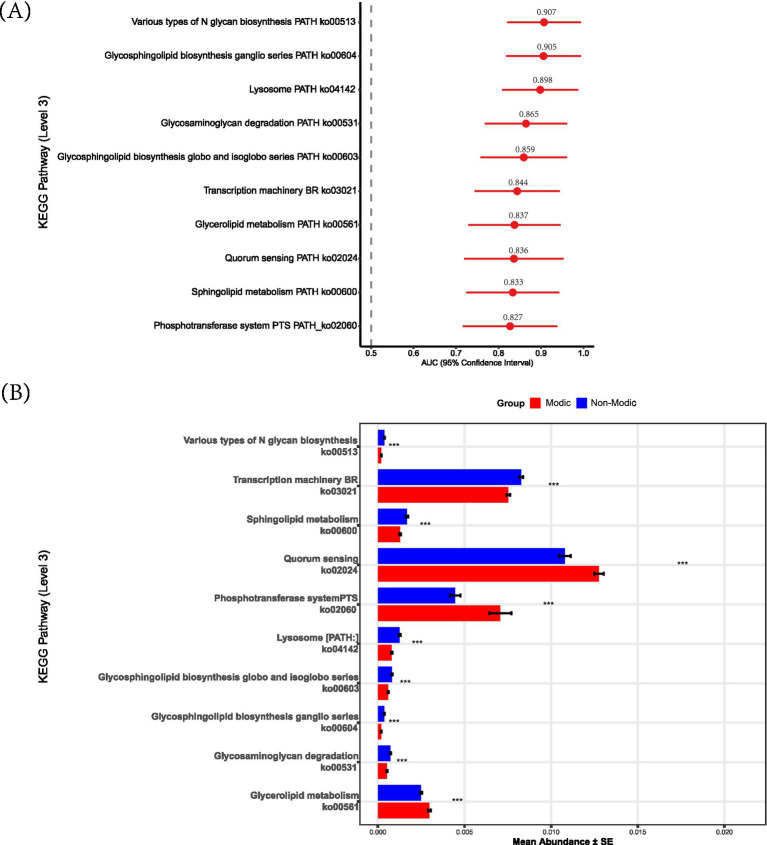
Discriminatory KEGG pathways between Modic and non-Modic groups. **(A)** AUC performance with 95% confidence intervals for the top 10 KEGG pathways. Pathways are ranked by descending AUC values from top to bottom. The dashed line indicates random classification (AUC = 0.5). **(B)** Abundance distribution of top 10 discriminatory KEGG pathways (level 3) identified by AUC analysis. Bars represent mean abundance ± SD (*N* = 56). Statistical significance markers: * *p* < 0.05, ** *p* < 0.01, *** *p* < 0.001.

To evaluate the diagnostic potential of these pathways, we conducted receiver operating characteristic (ROC) curve analysis on the 52 differentially abundant pathways. The top 10 pathways achieved AUC values ranging from 0.827 to 0.907 (Figure 8A). The abundance of these top 10 pathways differed significantly between the MCs and non-Modic groups (*p* < 0.001 for all; Figure 8B). Pathways such as Quorum sensing (ko02024), Glycerolipid metabolism (ko00561), and Phosphotransferase system *(PTS)* (ko02060) were markedly upregulated in MCs subjects (*p* < 0.001). Conversely, pathways involved in *Sphingolipid metabolism* (ko00600), Transcription machinery (ko03021), Glycosaminoglycan degradation (ko00531), and glycosphingolipid biosynthesis [e.g., Ganglio series (ko00604) and globo and isoglobo series (ko00603)] were significantly downregulated.

**Figure 9:**
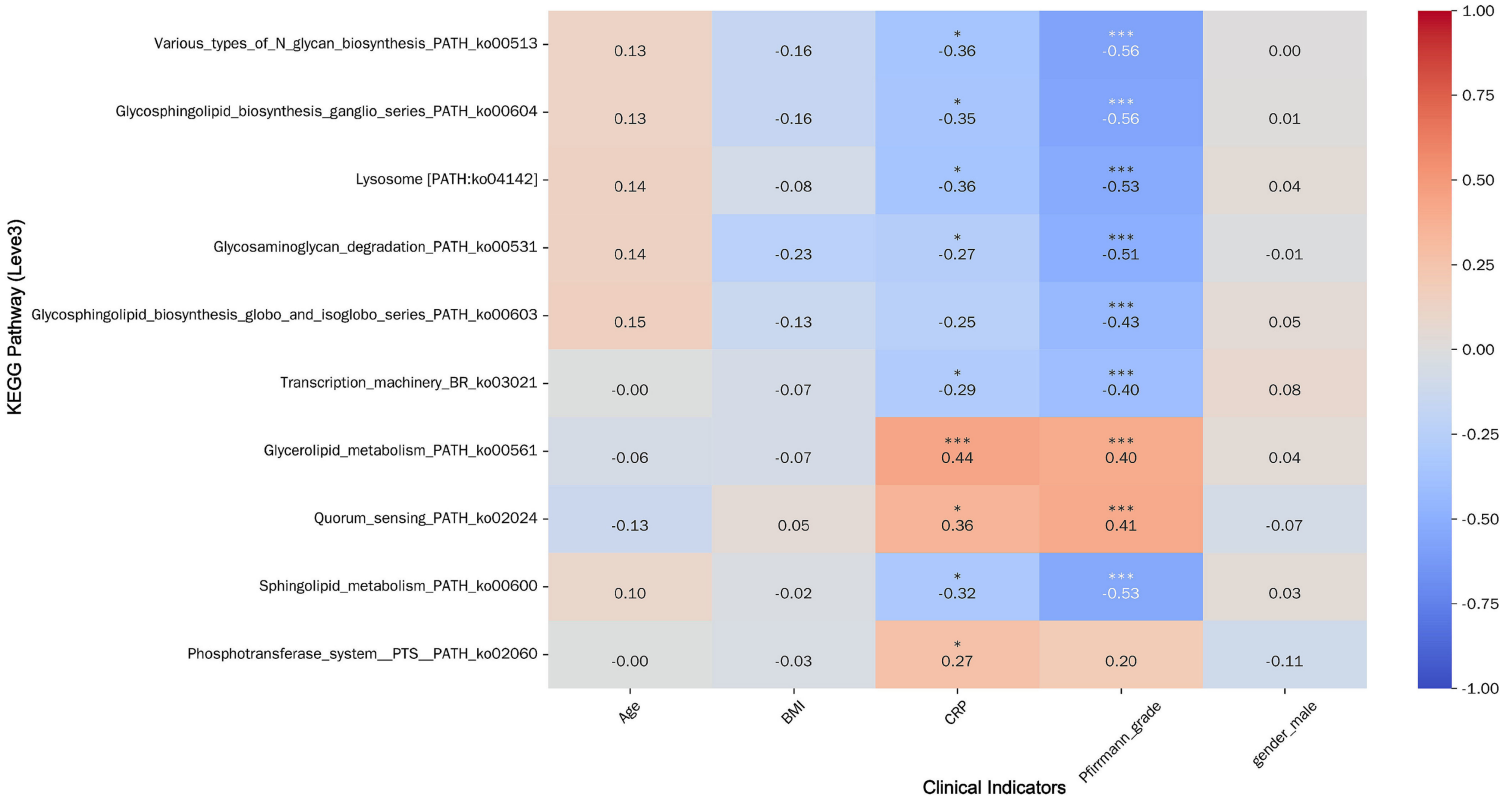
Heatmap of correlation between top 10 pathways by AUC value and clinical indicators. Pathways are displayed horizontally on the left, and clinical indicators are shown at the bottom. Color intensity reflects the strength of correlations (warm colors indicate positive correlations, cool colors indicate negative correlations). Statistical significance is denoted by asterisks above the correlation coefficients: * *p* < 0.05, ** *p* < 0.01, *** *p* < 0.001.

Correlation analysis with clinical variables indicated that CRP and Pfirrmann grade were significantly associated with most pathways (*p* < 0.05), except for specific glycosphingolipid and PTS pathways (Figure 9). In contrast, age, BMI, and sex showed no significant linear correlations with most metabolic pathways. Notably, both CRP and Pfirrmann grade exhibited significant positive correlations with *Glycerolipid metabolism* (ko00561) and *Quorum sensing* (ko02024) (*p* < 0.001)—pathways that were also upregulated in the MCs group. This consistency between clinical correlations and group-wise differential abundance strengthens the hypothesis that these microbial metabolic activities are biologically relevant to MCs pathogenesis.

## 4 Discussion

Our study demonstrates a significant association between gut microbial dysbiosis and Modic changes (MCs). Compared to healthy controls, individuals with MCs exhibited a significant reduction in gut microbial alpha diversity, particularly pronounced in those with inflammatory Modic Type 1 changes (MC1). Beta diversity analysis further confirmed distinct microbial community structures between individuals with MCs and controls. Specific bacterial taxa, including unclassified Parabacteroides and *Bacteroides uniformis*, demonstrated high discriminatory capacity for MCs. Additionally, shifts in microbial composition were significantly associated with MC severity grades, characterized by a decline in potentially beneficial taxa (e.g., *Bifidobacterium adolescentis*) and an enrichment of taxa implicated in pro-inflammatory processes (e.g., *Clostridium ramosum*, *Eubacterium hallii*). Metagenomic functional profiling revealed significant alterations in metabolic pathways, most notably an enrichment of quorum sensing and glycerolipid metabolism in individuals with MCs. These enriched pathways exhibited significant correlations with elevated serum inflammatory markers (CRP) and advanced disc degeneration, as determined by the Pfirrmann grading system.

The observed reduction in gut microbial diversity among individuals with Modic changes (MCs), particularly pronounced in Modic Type I, suggests a link between dysbiosis and vertebral endplate inflammation. This finding is consistent with accumulating evidence implicating gut microbiota dysbiosis in systemic inflammation, including musculoskeletal disorders (Zaiss et al., 2019; Aboushaala et al., 2024; Rajasekaran et al., 2024). The pronounced dysbiosis observed in Modic Type 1 patients – who exhibited more severe inflammatory profiles (mean serum CRP: 20.2 ± 11.0 mg/L) and advanced disc degeneration (62.5% with Pfirrmann grade V collapse) – may indicate that microbial imbalances either exacerbate or contribute to the inflammatory processes underlying MC I. This subtype is pathologically defined by active inflammation without adipose replacement (Jensen et al., 2008). In contrast, the lesser, non-significant reduction in alpha diversity observed in Modic Type 2 (Chao1 index, *p* = 0.065) might reflect a transition to a less inflammatory state characterized by adipose replacement. This differential pattern suggests a potential temporal or severity-dependent relationship between the degree of gut microbial dysbiosis and the progression of MC subtypes (Almeida et al., 2019).

Beyond these broad ecological shifts, we pinpointed specific microbial taxa implicated in MC pathogenesis. The identification of bacterial biomarkers, such as unclassified *Parabacteroides* and *Bacteroides uniformis*, with high discriminatory capacity for Modic changes (MCs) (AUC > 0.80), underscores the potential involvement of specific microbial taxa in MC pathogenesis. *Bacteroides uniformis*, which has been previously associated with anti-inflammatory effects in metabolic disorders, exhibited paradoxically high abundance in MC patients. This finding may indicate a context-specific pro-inflammatory or compensatory role within the vertebral endplate inflammatory microenvironment (Byndloss and Bäumler, 2018; Zmora et al., 2019a; Fabersani et al., 2021). This discrepancy underscores the complexity of microbiota-host interactions across different pathological states and necessitates further mechanistic investigation.

To move beyond associative links and identify microbial features predictive of disease severity, we applied a Random Forest machine learning model. This approach identified ten taxa as key biomarkers for MC severity. Among these, *Actinomyces oricola* was ranked as the top predictor (mean decrease in accuracy = 2.37), followed by *Eubacterium* sp. 36_13 (2.15). Compositional analysis across MC severity grades revealed a striking microbial shift: the abundance of the putative anti-inflammatory species *Bifidobacterium adolescentis* plummeted from 69.4% in Grade 1 to 29.8% in Grade 3 (*p* < 0.001) (Lim and Kim, 2017). Conversely, pro-inflammatory taxa such as *Clostridium* sp. TM06.18 and *Clostridium* sp. AF37.5AT, along with *Eubacterium* sp. 36_13, were significantly enriched in higher grades (*p* < 0.05). This progressive enrichment of *Clostridium* species and *Eubacterium* sp. with increasing MC severity aligns with their recognized capacity to produce pro-inflammatory metabolites, including secondary bile acids. These metabolites represent potential mediators influencing systemic inflammation and bone metabolism via the gut-bone axis (Ridlon et al., 2016; Guo et al., 2020). This inverse relationship suggests a shift in the gut microbial community from a protective to a pro-inflammatory state as MCs progress, potentially mediated through the production of pathogenic metabolites (Lavelle and Sokol, 2020). Notably, the abundance of *Faecalibacterium* sp. An192 remained stable across grades (5.6–6.0%, *p* > 0.05) despite its high predictive importance, implying its role may be defined by functional changes rather than population size (Louca et al., 2018). Spearman correlation analysis robustly supported these findings.

To delineate functional consequences underlying these taxonomic shifts, metabolic pathway analysis provided additional insights into the functional consequences of dysbiosis in Modic changes (MCs). Significant enrichment of quorum sensing and glycerolipid metabolism pathways in the MC group (*p* < 0.001) suggests enhanced microbial communication and lipid-related metabolic activity, which may contribute to systemic inflammation and bone marrow alterations (Papenfort and Bassler, 2016; Canfora et al., 2019). Quorum sensing, a mechanism regulating bacterial density-dependent behaviors, could amplify inflammatory signaling via the release of pathogen-associated molecular patterns (PAMPs) into the circulation, potentially impacting the vertebral endplate microenvironment (Whiteley et al., 2017). Similarly, alterations in glycerolipid metabolism may influence the production of pro-inflammatory lipid mediators, correlating with the elevated serum CRP levels observed in our cohort (*p* < 0.001) (Trauner et al., 2010). Conversely, the downregulation of pathways such as sphingolipid metabolism (*p* < 0.01) in MC patients is noteworthy. Given the established roles of sphingolipids in maintaining cellular homeostasis and immune regulation, this downregulation potentially signifies a loss of protective microbial functions (Bryan and Del Poeta, 2018; Hannun and Obeid, 2018). Critically, the depletion of microbial sphingolipids could impair intestinal barrier integrity and modulate systemic immune responses, a mechanism that may potentiate the inflammatory processes observed at the vertebral endplate (Li et al., 2022). This is highly consistent with the pro-inflammatory microenvironment that characterizes MCs.

Our results align with prior studies linking gut dysbiosis to systemic inflammatory and musculoskeletal disorders. For instance, gut microbial metabolites, such as short-chain fatty acids (SCFAs), have been shown to regulate bone homeostasis via immune modulation (Zaiss et al., 2019), a mechanism potentially pertinent to MCs in light of the observed metabolic pathway alterations. Similarly, Similarly, commensal gut microbiota can exert catabolic effects on skeletal homeostasis through immunomodulatory actions (Novince et al., 2017), supporting our observation of microbial community shifts associated with MC severity. However, despite studies investigating gut microbiota in osteoarthritis (Biver et al., 2019), direct evidence linking dysbiosis to MCs remains scarce. Consequently, our study represents a novel contribution to this underexplored area. Contrasting with some literature, our finding of high *Bacteroides uniformis* abundance in MC patients diverges from its established beneficial role in alleviating obesity-related inflammation and metabolic dysfunctions (Wang et al., 2019), suggesting disease-specific microbial adaptations or differing host responses in MCs. This divergence may stem from methodological differences, including the distinct disease contexts (metabolic disorders vs. musculoskeletal conditions) or variations in microbial sequencing techniques (e.g., 16S rRNA gene sequencing vs. the shotgun metagenomics employed herein). Furthermore, although no significant differences in gut microbial diversity were reported between intervertebral disc degeneration (IVDD) patients and controls (Rajasekaran et al., 2020), diversity reductions were specifically identified in our MC cohort. Our research extends beyond these prior works by integrating clinical imaging (Pfirrmann grade), inflammatory markers (CRP), and multi-omics data (microbiome and metabolomics), providing a more comprehensive analysis of the gut-MC axis. Furthermore, our metabolic pathway findings contribute significantly to understanding the functional roles of the microbiome in systemic inflammation. The increased activity of quorum sensing pathways in our MC cohort suggests enhanced microbial communication, a fundamental process for coordinating bacterial community behavior that is known to involve highly specific signal molecules and potential interspecies cross-talk, as detailed in studies of quorum sensing mechanisms (Wellington and Greenberg, 2019). Conversely, the decreased activity of glycosaminoglycan degradation pathways in our MC cohort contrasts with previously reported alterations in related pathways in osteoarthritis (Boer et al., 2019). This disparity potentially reflects disease-specific microbial contributions or differences in host tissue targets (articular cartilage vs. vertebral endplate). Collectively, these inconsistencies highlight the necessity for context-specific investigation of the microbiome in musculoskeletal disorders.

The principal innovation of our study resides in identifying distinct microbial and metabolic signatures linked to MC subtypes, directly addressing a critical knowledge gap concerning the gut-bone axis in spinal pathologies. By demonstrating correlations between microbial shifts, metabolic pathway alterations, and clinical markers, our findings establish a foundation for developing potential diagnostic tools and therapeutic targets, extending beyond the broader exploratory associations reported in previous studies on the gut microbiota in spinal or musculoskeletal conditions (Szychlinska et al., 2019; Rajasekaran et al., 2020; Geng et al., 2023). While these earlier investigations primarily focused on establishing links between microbial composition and degenerative disc diseases or associated inflammatory states, our work provides a mechanistically focused investigation specifically targeting Modic changes.

Collectively, the observed taxonomic shifts and functional pathway alterations (e.g., quorum sensing enrichment, sphingolipid metabolism downregulation) point to several plausible mechanistic links between gut dysbiosis and MCs. Based on the multi-omics signatures and clinical correlations reported above, we propose three testable mechanistic pathways: several plausible mechanisms may explain the observed associations between gut dysbiosis and MCs. First, reduced microbial diversity and depletion of potentially beneficial taxa (e.g., *Bifidobacterium adolescentis*, reduced from 69.4 to 29.8% at Grade 3) may impair production of anti-inflammatory metabolites like SCFAs, which critically modulate systemic immune responses (Parada Venegas et al., 2019). This reduction could exacerbate vertebral endplate inflammation, particularly in inflammatory Modic Type I evident on MRI (Modic et al., 1988). Second, the enrichment of quorum sensing pathways in MC patients implies heightened bacterial cell-to-cell communication and potential coordination of virulence traits (Abisado et al., 2018). This enhanced microbial signaling may amplify systemic inflammatory responses through the release of specific metabolites or via host immune activation, ultimately contributing to alterations in the vertebral microenvironment. Third, upregulated glycolipid metabolism may contribute to lipid accumulation or inflammatory mediator production, aligning with the adipose replacement characteristic of Modic Type II and correlating with elevated CRP levels in our cohort (Schoeler and Caesar, 2019).

These findings support the emerging gut-bone axis concept (Ohlsson and Sjögren, 2018), wherein the gut microbiota influences skeletal health through immune and metabolic pathways. Our data suggest MCs represent a specific manifestation of this axis, with dysbiosis potentially amplifying local inflammation and degeneration in vertebral endplates. However, we emphasize that these interpretations are correlative, and causality remains unestablished. Alternative explanations, such as systemic inflammation or pain-related lifestyle changes driving dysbiosis, cannot be excluded. Therefore, we present these as testable hypotheses grounded in our data, avoiding definitive mechanistic claims, and call for experimental studies to validate the proposed pathways.

Our study has several limitations that warrant consideration. First, the sample size (*n* = 31 MC patients, *n* = 25 controls) may limit the statistical power to detect subtle microbial differences, particularly for low-abundance taxa or pathways with smaller effect sizes. This could lead to an underestimation of certain associations and restricts the generalizability of findings to broader populations. Second, the case–control design precludes causal inferences regarding the relationship between gut dysbiosis and MC development. It remains unclear whether microbial alterations precede MC onset or result from associated inflammation, pain, or lifestyle factors (e.g., diet, reduced activity) (Belkaid and Hand, 2014). Third, reliance on stool samples for microbiome analysis may not fully reflect microbial dynamics at the vertebral endplate, where loc microbial infiltration has been hypothesized as a contributing factor in MCs (Albert et al., 2013). Finally, the sequencing depth of our metagenomic analysis may have missed low-abundance taxa or functional genes, potentially underrepresenting the full microbial contribution to MC pathology. These limitations affect the interpretation of our results by introducing potential biases and constraining external validity. Nevertheless, our robust statistical adjustment, integration of clinical and multi-omics data, and focus on MC-specific signatures provide a strong basis for hypothesis generation and future validation, without diminishing the study’s overall value.

Despite these limitations, our study robustly identifies novel associations between gut microbial/metabolic signatures and MCs, providing a foundational hypothesis-generating framework for this underexplored field. Based on our findings and limitations, we propose several specific directions for future research. First, longitudinal studies with serial microbiome sampling and MRI assessments are essential to determine the temporal relationship between gut dysbiosis and MC progression, clarifying whether microbial changes precede or follow disease onset. Second, experimental studies using animal models (e.g., germ-free mice colonized with MC patient microbiota) could test the causal impact of specific taxa or metabolites on vertebral endplate inflammation and degeneration. Third, larger, multicenter studies in diverse populations are needed to validate our identified biomarkers (e.g., unclassified_Parabacteroides) and assess their diagnostic utility across ethnic and environmental contexts, addressing the sample size limitation. Fourth, integrating local microbial profiling (e.g., endplate biopsies) with gut microbiota data could elucidate whether systemic dysbiosis mirrors local microbial changes in MCs. Fifth, while we controlled for major systemic comorbidities and medications, we did not quantitatively assess psychological stress, which is a known modulator of the gut microbiome. Future studies incorporating validated stress questionnaires would provide a more comprehensive view of the factors influencing the gut-bone axis in MCs (Wang et al., 2025). Finally, intervention studies targeting gut microbiota (e.g., probiotics, prebiotics, or dietary modifications) should explore their potential to modulate inflammation and improve clinical outcomes in MC patients, translating mechanistic insights into practical applications (Davani-Davari et al., 2019; Liu et al., 2019; Markowiak-Kopeć and Śliżewska, 2020). Such interventions are supported by recent research highlighting the therapeutic potential of microbial modulation for systemic inflammation and related health conditions (Rinninella et al., 2019; Zmora et al., 2019b; Vinderola et al., 2022).

## 5 Conclusion

In conclusion, our study demonstrates a significant association between gut microbial dysbiosis, characterized by reduced diversity, distinct bacterial biomarkers, and altered metabolic pathways, and Modic changes in patients with low back pain. These findings offer novel evidence for the role of the gut-bone axis in spinal pathology, directly addressing the research question of whether microbial alterations contribute to MC development and progression.

## Data Availability

The raw sequencing data generated in this study have been deposited in the NCBI Sequence Read Archive (SRA) under the accession number PRJNA1314852.
