# Supplementary material for: Gut microbiome dysbiosis implicates the gut-bone axis in Modic changes: a metagenomic case–control study
Source: Front Microbiol. 2025 Dec 15;16:1702357. doi: 10.3389/fmicb.2025.1702357 (PMC12747672; doi:10.3389/fmicb.2025.1702357)
Supplement: Supplementary file 1 [file Supplementary_file_1.docx]

Supplementary Material

# Supplementary Figures


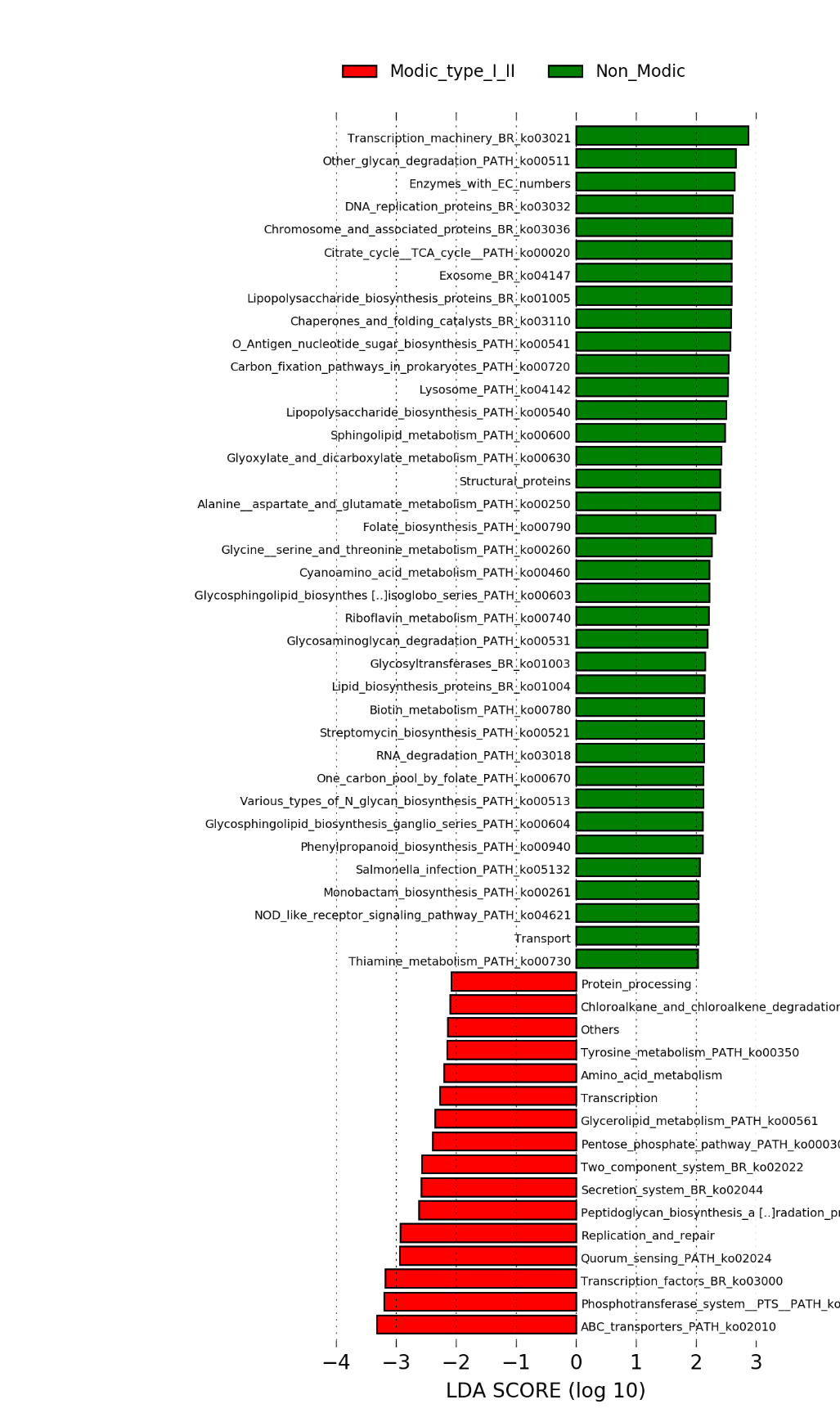


**Supplementary Figure 1.** To identify functional biomarkers with significant differences between groups, we first employed the rank-sum test to detect differentially abundant functions across different groups. This was followed by linear discriminant analysis (LDA) for dimensionality reduction and evaluation of the effect size of these differential functions, resulting in the LDA score. For this LEfSe analysis, the significance thresholds were set as follows: LDA score > 2.0 and p-value < 0.05. The bar chart below shows the distribution of LDA scores for the differentially abundant functions between groups.
